# Infertility Stigma: A Qualitative Study on Feelings and Experiences of Infertile Women

**DOI:** 10.22074/IJFS.2021.139093.1039

**Published:** 2021-06-22

**Authors:** Mahboubeh Taebi, Nourossadat Kariman, Ali Montazeri Ph.D.4, Hamid Alavi Majd

**Affiliations:** 1 Department of Midwifery and Reproductive Health, School of Nursing and Midwifery, Shahid Beheshti University of Medical Sciences, Tehran, Iran; 2 Department of Midwifery and Reproductive Health, Isfahan University of Medical Sciences, Isfahan, Iran; 3 Department of Midwifery and Reproductive Health, Midwifery and Reproductive Health Research Center, School of Nursing and Midwifery, Shahid Beheshti University of Medical Sciences, Tehran, Iran; 4 Health Metrics Research Centre, Iranian Institute for Health Sciences Research, ACECR, Tehran, Iran; 5 Department of Biostatistics, School of Allied Medical Sciences, Shahid Beheshti University of Medical Sciences, Tehran, Iran

**Keywords:** Female Infertility, Infertility, Stigma, Qualitative Study

## Abstract

**Background:**

Infertility stigma is a phenomenon associated with various psychological and social tensions especially for women. The stigma is associated with a feeling of shame and secrecy. The present study was aimed to explore the concept of infertility stigma based on the experiences and perceptions of infertile women.

**Materials and Methods:**

This qualitative conventional content analysis study was conducted in Isfahan Fertility and Infertility Center, Iran. Data were collected through in-depth interviews with 17 women who had primary infertility. All the interviews were recorded, transcribed and analyzed according to the steps suggested by Graneheim and Lundman. The Standards for Reporting Qualitative Research (SRQR) checklist was followed for this research.

**Results:**

Eight hundred thirty-six initial codes were extracted from the interviews and divided into 25 sub-categories, 10 categories, and four themes. The themes included “stigma profile, self-stigma, defensive mechanism and balancing”. Stigma profile was perceived in the form of verbal, social and same sex stigma. Self-stigma was experienced as negative feelings and devaluation. Defensive mechanism was formed from three categories of escaping from the stigma, acceptance and infertility behind the mask. Two categories; empowered women and pressure levers, created a balancing theme against the infertility stigma.

**Conclusion:**

Infertile women face social and self-stigma which threatens their psychosocial wellbeing and self-esteem. They use defensive response mechanisms and social support to mitigate these effects. Education focused on coping strategies might be helpful against infertility stigma.

## Introduction

Infertility and subfertility affect a significant proportion of human beings (1). Infertility is defined as failure to achieve clinical pregnancy after 12 months of regular unprotected sexual intercourse. In general, 8 to 12% of couples of reproductive age suffer from infertility worldwide (2). According to a World Health Organization report, more than 10 percent of women are affected by infertility (1). In addition to the medical problems, infertility can cause numerous personal and social problems. It can be seen as a developmental crisis (3). Infertility can have damaging social and psychological consequences from exclusion and divorce to social stigma that leads to isolation and psychological distress (4).

Although infertility affects both sexes equally, it is women who are most frequently blamed (5). This causes infertile women to feel guilty and threatens their selfesteem. Thus, infertile women experience greater psychological stress than infertile men, and they are often stigmatized for being infertile and being childless (6). Many women experience infertility as a stigma. Although it seems that infertility stigma is likely to be greater in developing countries, infertility has been stigmatized in both developed and developing countries (7, 8).

Infertility stigma is associated with the feeling of shame and secrecy (9, 10). Stigma is defined as a negative feeling of being different compared to others in society and being contrary to social norms (11). If infertility is ex-perienced as a stigma, it has the potential to deprive the infertile person of social support and cause depression, anxiety and stress (4, 12), feelings of guilt (13) and relationship problems (5). It may also cause psychological disturbance, decreased self-esteem and self-efficacy, and a tendency toward self-stigma (14). Infertility stigma and its related social pressures influence all the dimensions of women’s lives and well-being. Qualitative studies can provide more in-depth understanding of infertility stigma and can help develop more effective interventional strategies. Due to the limited number of qualitative studies in this field, this study was conducted to explore the feelings and experiences of infertile women regarding infertility stigma.

## Materials and Methods

### Design and data collection

This study is a qualitative content analysis conducted in Isfahan Fertility and Infertility Center, Isfahan, Iran. Women with known infertility who were under infertility treatments participated in the study. The inclusion criteria consisted of having primary female infertility and absence of any psychological disorders. Participant’s likelihood of withdrawing from the study was considered as the only exclusion criterion. Purposive sampling was carried out from 2019 to 2020 to ensure maximum variation in terms of age, education, occupation and infertility duration. The present article adheres to the EQUATOR guidelines of reporting research using the Standards for Reporting Qualitative Research (SRQR) checklist (15).

Twenty-one women were asked to participate in the study of which four refused because they were not interested in the subject or had a busy schedule.

A private and comfortable room was provided in the center and women were free to choose the place of the interview. All the participants preferred the private room in the center for their interviews. Semi-structured face-toface interviews were conducted to assess the perceptions of women about infertility stigma. The researcher used interviewing skills to provide an intimate and comfortable atmosphere for the participants and helped them express their experiences of infertility stigma. All the interviews were conducted by the first author (M.T); a researcher in the field of infertility, and qualitative research. Two pilot interviews were conducted to improve the question guide. Interviews were organized based on the research question and the data from the literature review. The interviews began with open-ended questions such as “How did you feel about your infertility?”, “How did infertility affect your life?", and “Did you experience any special treatment because of your infertility? Probing questions such as “How?”, “What do you mean?” and “Please explain more on this issue” were asked to elicit further information. With the progress of the study, some direct questions were added to the interviews such as “Have you experienced labeling because of your fertility problem?” and “Do you feel any social pressure because of your fertility problem?” 

In-depth interviews were continued until data saturation was reached; meaning that no new meaning unit was extracted from the interviews. The duration of the interviews varied between 30 to 45 minutes. All the interviews were voice recorded and then transcribed as soon as possible after the interview. The feelings and emotions of the participants during the interviews also were noted.

### Data analysis and trustworthiness

Conventional content analysis using the Graneheim and Lundman method was applied throughout the data collection (16). Transcription, analysis and coding of each interview was done before the beginning of the next interview. The contents of the interviews were completely transcribed. Transcripts were read several times to gain understanding and identify initial categories of meaning and codes. Codes, sub-categories, categories and themes were derived from the transcripts. Combinations of related initial codes were labeled to form sub-categories and categories. Finally, the latent meaning of the text and the main themes were developed until consensus between the researchers was reached and the concept of stigma in infertile women was fully described.

Trustworthiness of the data was determined as suggested by Guba and Lincoln (16). To establish internal validity, transcripts were reviewed immediately after they were made. Adequate time was assigned to data collection, and the first author had prolonged engagement with the study subjects. The transcripts and codes were shared with two participants to ensure congruence between their experiences and the study findings (member check). For dependability of the data, external reviewers, who were not members of the research team and were familiar with qualitative studies, approved the units of meaning, codes, subcategories, categories, and themes and made suggestions that were considered in the final analysis. The external reviewer was asked to extract meaning units and initial codes of two interviews. Then the percentage of agreement between initial codes was calculated, which showed inter coder reliability (ICR) was more than 90% (17). 

Finally, to establish the external validity that demonstrates transferability, the authors provided a detailed description of the participants and their experiences, and the research design. In addition, selected interviews, along with codes and categories, were shared with two infertile women other than the participants and they agreed that these codes represented their real experiences (18).

### Ethical consideration

All participants were informed of the study purpose and assured of the confidentiality of their data and their voluntary participation. All the interviews were conducted in a private and comfortable room. Informed written consent was obtained from the participants that included consent to recording their interview. The Researc and Ethics Committee of the Shahid Beheshti University of Medical Sciences approved the study (Approval ID: IR.SBMU.RETECH.REC.1397.310).

## Results

Seventeen infertile women participated in the study. Although data saturation was reached after 14 interviews, the authors conducted three more interviews to ensure saturation of the data. The mean age of the women was 32.88 years. The average duration of infertility was 4.25 years. The characteristics of the participants are shown in Table 1. 

**Table 1:** The characteristics of the participants (n=17)


Characteris‌tics of the participants	n (%)

Age (Y) [32.88 ± 4.82]^*^	
Less than 25	4 (23.5)
25-35	9 (53)
More than 35	4 (23.5)
Infertility duration (Y) [4.25 ± 3.71]^*^	
Less than 5	9 (53)
5-10	6 (35.3)
More than10	2 (11.7)
Education	
Less than diploma	3 (17.6)
Diploma	6 (35.3)
Academic	8 (47.1)
Employment s‌tatus	
Housewife	12 (70.6)
Employed	5 (29.4)


*; Mean ± SD.

836 initial codes were extracted from the interviews and categorized into 25 sub-categories, 10 categories and four main themes. The four main themes that emerged during data collection were identified as: stigma profile, self-stigma, defensive mechanism and balancing (Table 2)

### Theme 1: Stigma profile

The experiences of infertile women showed they have perceived infertility stigma. Stigma profile was experienced as verbal stigma, social stigma and same sex stigma.

### Verbal stigma

One of the distressful behaviors mentioned by all the participants was verbal stigma in the form of sarcasm, humiliation, and use of offensive terms for infertility by acquaintances.

A 32-year-old participant, with secondary education, housewife, 10-year infertility duration said:* “The old people say that if someone doesn’t have a child, their house is empty. They call them [OjaghKoor] (a humiliating word that means the couple’s house is cold and spiritless). Some say to me “how incapable you are that you could not bring a child for your husband.”*

**Table 2:** The theme, categories and subcategories of the infertility stigma concept


Themes	Categories	Sub-categories

S‌tigma profile	Verbal s‌tigma	Sarcasm and humiliation
		Curiosity
	Social s‌tigma	Discrimination
		Negative burden of infertility
	Same sex s‌tigma	Women agains‌t women
		Sexism by women
Self-s‌tigma	Negative feelings	Bitter feeling of infertility
		Sadness and regret
		Fear and concern
	Devaluation	Incomplete woman
		Transformation of values
		Low self-es‌teem
		Low self-efficacy
Defensive mechanism	Escaping from s‌tigma	Looking for someone to blame
		Jus‌tifying the infertility
	Acceptance	Getting along with the problem
		Unchangeable fate
	Infertility behind the mask	Secrecy
		Silence
Balancing	Empowered women	Resilience
		Optimism
	Pressure levers	Supportive/Unsupportive husband
		Peer support
		Supportive family
		Pressure from husband’s family


Most participants encountered a huge number of curious questions from their acquaintances such as *why haven’t you had children yet? Do you have a problem or does your husband have any problems? *These questions were considered offensive and annoying in the eyes of the women. 

### Social stigma

The attitude of community members and their negative views toward infertility were pointed out by most participants.

*“From their type of look I can understand what they are thinking. Infertility does not bother me at all, but their looks do.”* (34-year-old participant, with bachelor’s degree, accountant, 5-year infertility duration) 

*“People think differently about you. It looks like you are different” *(25-year-old participant, with primary school degree, housewife, 8-year infertility duration)

Most participants were reluctant to use the term infertility. They usually referred to it as “the issue”, “the problem”.

*"I do not like the word of infertility at all. I do not think it is a good word at all.”* (35-year-old participant, with diploma degree, housewife, 9-year infertility duration)

### Same sex stigma

Most participants complained about being labeled by other women. 

*“When my mother-in-low introduces me to others, she says: she is my daughter-in-law, she is in our family for 13 years but still has no children. Please pray for her. She wants to hurt me; she wants to say that the problem is from my side.”* (30-year-old participant, with middle school degree, housewife, 9-year infertility duration)

Some participants said that: *“They are women themselves, they should understand other women’s problems, and they have daughters themselves.” *(33-year-old participant, with doctoral degree, 1year infertility duration)

Some women experienced different types of sexism from other women. A participant said: *“The men in the family have more empathy with me than the women. My father-in-law is very kind and never asks a question to bother me, but women like their son in law more.”* (32-year-old participant, with diploma degree, 1year infertility duration) 

### Theme 2: Self-stigma

Sometimes infertile women internalize the process of stigma. We could identify at least two elements that contributed to self-stigma: negative feelings and devaluation. 

### Negative feelings

The experiences of some of the participants indicated their suffering and sadness. Repeated questions from acquaintances would lead to psychological distress. The negative feelings that these infertile women experienced were expressed as bitterness, sadness and anxiety.

*“I think that infertility is a disaster. The disease itself could be treated, but what happens in our society and the way that others treat you, it is really bad. The fact that everybody believes that it is your fault.”* (30-year-old participant, with middle school degree, housewife, 5-year infertility duration)

Infertility and the outcomes surrounding it, including the possibility of separation and remarriage of the husband, occupied the women’s minds, and many of them, despite having the support of their husbands, were afraid that their marital lives would collapse. The idea that not having a child would make their husband bored with them and that they might look for someone else always bothered them.

### Devaluation

Participants believed that infertility was the reason for their incompleteness and defect. Consequently, they had a feeling of inferiority.

*“I always think that, because I cannot get pregnant, cannot have children, I am lower than others. This idea really bothers me.” *(34-year-old participant, with primary school degree, housewife, 10-year infertility duration)

Sometimes these feelings of inferiority made them transform their beliefs, and personal values and led to deterioration in their self-esteem.

*“My cousin was divorced when she didn’t get pregnant after 13 years. I supported her. I used to say that having a child is not the most important role of a woman. I did not know that I would have the same fate. "*(26-year-old participant, with bachelor’s degree, housewife, 2-year infertility duration)

*“I’m not comfortable at parties at all. I don’t have a good feeling. My self-esteem has really decreased. I don’t want to be among others. I feel like I’m boring in comparison to them.”* (35-year-old participant, with diploma degree, housewife, 9-year infertility duration)

These negative emotions reduced women's self-efficacy, and they were not able to control their feelings and emotions.

*“I became very sensitive. My brother's wife became pregnant. I did not want to see her during pregnancy at all.”* (37-year-old participant, with doctoral degree, 14-year infertility duration).

### Theme 3: Defensive mechanism

Infertile women unconsciously employed defensive response mechanisms when they encountered the stress of infertility stigma to protect themselves from psychosocial harm. Women used a combination of defensive response mechanisms, such as escaping from stigma; acceptance; and infertility behind a mask.

### Escaping from stigma

Avoiding acceptance of their infertility, and irrational justifications for infertility were some of the mechanisms that participants used to escape from being labeled.

*“Now that we are going to herbal therapy, it turns out that my husband is weak! I told my mother-in-law, now you see it was not my problem, but your son is weak. *(29-year-old participant, with diploma degree, housewife, 2-year infertility duration).

### Acceptance

Over time, as the duration of their infertility lengthened, some participants considered infertility undeniable and tried to face it rationally and accept it as their fate.

*“It could not be denied. But it has become really normal to me and I am trying to get along with it. My grandma always used to say, the life is not always in our favor, so be patient and satisfied by what you get” *(37-year-old participant, with doctoral degree, 14-year infertility duration)

### Infertility behind the mask

Most participants were hiding their infertility from their family and relatives, especially their husband’s family. By remaining silent about their fertility problem, participants escaped the judgments and pitiful looks of others.

*“I don’t like anybody to know anything about this at all. I don’t like to be looked on with pity. Whenever I’m asked when you’re going to have children, I’d say I don’t have time for children because I go to work. I come to the center for treatment, but I don’t tell anybody” *(42-year-old participant, with master’s degree, consultant, 3-year infertility duration)

These participants always mentioned excuses such as working and being busy, studying or pretending to have decided not to have children when encountering curious questions from others.

### Theme 4: Balancing

Infertile women used various factors to balance the psychological damage resulting from their perceived infertility stigma. This balancing was sub-divided into two categories; empowered woman and pressure levers.

### Empowered woman

Women endured and managed stressful relationships using a sense of humor, modifying relationships, and ignoring the judgment of others to protect against the psychological pressure caused by infertility stigma.

*“I turn it into fun, now. I say that my child doesn’t like me to be his/her mom. He/she would come whenever he/she wants. I won’t let them continue.”* (32-year-old participant, with diploma degree, housewife, 1year infertility duration)

By performing artistic, social, and athletic activities, women tried to avoid negative thoughts and eliminate the pressure of stigma, so they could bring balance into their lives.

*“I always want to make others aware. I even have a page on Instagram and I give information anonymously. It is more for giving awareness to the society. These activities amuse me in a way and are also good for my spirit.”* (34 year-old participants, with bachelor’s degree, accountant, 5-year infertility duration)

### Pressure levers

There are factors in the lives of participants that act as positive or negative levers and modify the pressure of infertility stigma. Interviews showed that infertile women received emotional support from various sources including their husbands, families, peer groups, and, in a limited number of cases, their friends. According to most participants, husbands were the most important source of emotional support.

*“My husband has said that the problem is with him, not me. He says all of this without putting any pressure on me.” *(32-year-old participant, with diploma degree, housewife, 1-year infertility duration) 

*“In response to others, my husband says that I know myself when is the right time to have a child. Right now, my life is good, I don’t need children now.” *(26-year-old participants, with bachelor’s degree, employee, 2-year infertility duration)

On the other hand, experiences of some participants showed that the behavior of their husband was not supportive, but, on the contrary, it was the source of tension for them. 

*“I said now that I have this problem, we can go and get a child from the orphanage, my husband objected, and he said I want a child of my own, even with another woman.”* (33-year-old participant, with diploma degree, housewife, 4-years infertility duration) 

Some participants mentioned that it is hard for others to comprehend what infertile women are going through. They believed that only women with the same problem could understand them. 

*“I would like to talk with people who are similar to me. When I talked with this friend of mine, who had adopted a child, I felt really good. We could understand each other pretty well. I was very happy when I came home after meeting her. I did the house works; I liked to put on makeup.” *(34-year-old participant, with primary school degree, housewife, 10-years infertility duration)

Some participants identified their family as a source of support. 

*“My family comforts me a lot. They say do not have stress. Everything is going to be alright.”* (34-year-old participant, with diploma degree, housewife, 4-years infertility duration)

Most participants cited their husband's family as a source of tension and stigma. Spousal family pressure for remarriage or divorce was one of the concerns of the infertile women.

*“My husband’s sister tells him, think for yourself while you are young. Go get remarried.”* (25-year-old participant, with primary school degree, housewife, 8-year infertility duration)

*“They say we want grandchildren. Why don't you do something? They ask which one of you is to blame for infertility?”* (36-year-old participant, with diploma degree, housewife, 1-year infertility duration)

## Discussion

The present study is one of the few studies that focuses on the perceptions and experiences of female infertility stigma. The research showed that the concept of infertility stigma was perceived as verbal, social and same sex stigma. Self-stigma was experienced as negative feelings, and devaluation. In contrast, women used defensive mechanisms in the form of escaping from stigma, acceptance and infertility behind the mask. They try to make a balance between the sense of empowerment and pressure levers.

The participants stated that they had been verbally humiliated by their acquaintances, being called sterile, issueless and fruitless. Other studies have also mentioned verbal sarcasm and using terms such as hollow, fruitless tree, dried tree and barren land (9, 12, 19). Curious questions from acquaintances were one of the concerns of infertile women that could threaten their mental health and could be associated with a wide range of psychological damages such as anxiety, depression and low self-esteem (13, 20, 21).

Social stigma referred to a situation in which infertile women would face discrimination from others; a different and compassionate look which was torturous to them. Mumtaz. et al stated that women perceived more stigma than men and that being stigmatized was more painful than being infertile (22). Furthermore, most of the participants did not like the term “infertile”. Psychologists believe that for such people, titles and labels should be used that do not imply a flaw; like using child free instead of childless (23).

Other women were the most considerable source of stigma. It seems that sometimes women are acting against women. A study in Niger showed that mostly women were the target of verbal and physical stigma from the women of their husband’s family (24). In most societies, even advanced ones, having a child of your own is considered a great privilege (25). Motherhood and having children is the only way for women to raise their standing in the family and the society (26). In traditional societies motherhood is one of the important roles of women and those who are not capable of performing this role are powerless in the eyes of other women and would be humiliated (25).

According to interviews, women might internalize the stigma and see themselves lower than other women. These women usually lose their self-esteem and are suffering from social isolation. Feelings of shame and inferiority (27, 28), worthlessness and losing control, social isolation and decreased self-esteem (5, 29, 30) have been reported in other studies. Furthermore, women stated that infertility could threaten their marriage, this has been reported in other studies too (5, 27). Fear of divorce and separation has also been reported in Asian and African societies (5, 7, 9, 24, 31).

Goffman suggests that the individual sometimes initiates a process of stigmatization inside themselves - internal or self-stigmatization (11). Self-stigma refers to negative attitudes created in individuals by themselves due to the conditions they have been put through. One of the factors destabilizing individual identity is self-stigma which seems to affect their self-efficacy (32).

People do not react similarly to stigma. Women used defensive mechanisms against the tensions caused by infertility stigma. The most important of these were hiding the infertility and infertility behind the mask. Silence and hiding were reactions that have been reported in other studies too (33, 34). Goffman suggests that the first strategy for confronting stigma is hiding it. Thinking that the stigmatized person will not be accepted they try to reduce the intensity of the stigma by hiding the problem (11). However, it must be considered that, when individuals hide their problem, they end up facing the problem alone, which makes them more anxious. They may also use inefficient coping strategies. The infertile women’s fear that their secret might be revealed is likely to increase tension, feelings of guilt and sadness, and leave them open to psychosocial pressures (5, 8, 35).

All the women, regardless of age, educational level or employment status, had experienced forms of stigma. However, empowered women, regardless of education and employment, were more successful in balancing the psychological outcomes of infertility stigma. Kabeer mentioned that self-respect, self-efficacy and psychological health could be improved by empowering women (36). Therefore, the care team should consider providing coping strategies to women suffering from infertility stigma.

Women mentioned some negative and positive sources that could help them to adjust to the pressures of infertility stigma. The most important source of support was their husbands. The husband played the most important role in defending his wife against the verbal and behavioral pressures of others, especially the in-laws. Results of a study in Australia also showed that a woman’s husband and mother were the strongest, and the mother-in-law the weakest source of support for infertile women (35). In-laws were one of the pressure levers also mentioned in other studies (5, 6) and could be one of the main sources of stigma for infertile women.

One of the women’s strategies for creating balance was communicating with other infertile women. Peer groups have been mentioned as an important source of support for women with fertility problems. Improving social relationships through the support of their peers could increase fertility-related quality of life (37). Peer support has a crucial role in therapeutic services, that should be considered by healthcare providers (38). This can complete the management of infertility and add mental health perspectives to formal treatments.

People make decisions about their problems according to their experiences (39), so interviewing women about their experiences of infertility stigma is valuable intself. The interviewer has a long history of working with women suffering from fertility problems as a faculty member of the midwifery and reproductive health department in the university. She introduced herself fully to the participants. The familiarity of the researcher with the subject of the study and cultural context might have helped participants to express their experiences and feelings better. This could be a strength of the present study. The present study is one of the few qualitative studies that have undertaken an in-depth investigation of infertile women’s experiences of infertility stigma.

Although the qualitative nature of the study means that its findings are relatively context dependent, they are likely to be generalizable to similar patient groups in similar settings. A limitation of the study is that the experiences of women who were infertile but had not been referred for treatment were not evaluated. This study presents a clear picture of infertility stigma and could be a springboard for further research related to infertility. It could also be used for developing protocols for psychological and counseling interventions appropriate for infertile women.

## Conclusion

Infertile women confront different forms of stigma that can lead to devaluation and self-stigma. On the other hand, women use different defensive mechanisms and try to make a balance between a sense of empowerment and pressure levers. Health personnel who provide services to infertile women should be aware of the stigma experienced by these women and its influences on their well-being. Education focused on coping strategies might be helpful against stigma. 

## References

[1] WHO Infertility is a global public health issue.

[2] Wasilewski T, Łukaszewicz-Zając M, Wasilewska J, Mroczko B (2020). Biochemistry of infertility. Clin Chim Acta.

[3] Datta J, Palmer MJ, Tanton C, Gibson LJ, Jones KG, Macdowall W (2016). Prevalence of infertility and help seeking among 15 000 women and men. Hum Reprod.

[4] Slade P, O'Neill C, Simpson AJ, Lashen H (2007). The relationship between perceived stigma, disclosure patterns, support and distress in new attendees at an infertility clinic. Hum Reprod.

[5] Hasanpoor-Azghady SB, Simbar M, Vedadhir AA, Azin SA, AmiriFarahani L (2019). The social construction of infertility among iranian infertile women: a qualitative study. J Reprod Infertil.

[6] Fu B, Qin N, Cheng L, Tang G, Cao Y, Yan C (2015). Development and validation of an infertility stigma scale for chinese women. J Psychosom Res.

[7] Karaca A, Unsal G (2015). Psychosocial problems and coping strategies among Turkish women with infertility. Asian Nurs Res (Korean Soc Nurs Sci).

[8] Greil AL, Slauson-Blevins K, McQuillan J (2010). The experience of infertility: a review of recent literature. Sociol Health Illn.

[9] Fledderjohann JJ (2012). 'Zero is not good for me': implications of infertility in Ghana. Hum Reprod.

[10] Pacheco Palha A, Lourenco MF (2011). Psychological and cross-cultural aspects of infertility and human sexuality. Adv Psychosom Med.

[11] Goffman E (2009). Stigma: notes on the management of spoiled identity.New York.Simon and Schuster.

[12] Carter J, Applegarth L, Josephs L, Grill E, Baser RE, Rosenwaks Z (2011). A cross-sectional cohort study of infertile women awaiting oocyte donation: the emotional, sexual, and quality-of-life impact. Fertil Steril.

[13] Donkor ES, Sandall J (2007). The impact of perceived stigma and mediating social factors on infertility-related stress among women seeking infertility treatment in Southern Ghana. Soc Sci Med.

[14] Sternke EA, Abrahamson K (2015). Perceptions of women with infertility on stigma and disability. Sex Disabil.

[15] O'Brien BC, Harris IB, Beckman TJ, Reed DA, Cook DA (2014). Standards for reporting qualitative research: a synthesis of recommendations. Acad Med.

[16] Guba E, Lincoln Y (1981). Effective evaluation: improving the usefulness of evaluation results throgh responsive and naturalistic approaches.

[17] O’Connor C, Joffe H (2020). Intercoder reliability in qualitative research: debates and practical guidelines. Int J Qual Methods.

[18] LoBiondo-Wood G, Haber J (2006). Nursing research: methods and critical appraisal for evidence-based practice.

[19] Dyer SJ, Abrahams N, Hoffman M, van der Spuy ZM (2002). Men leave me as I cannot have children: women's experiences with involuntary childlessness. Hum Reprod.

[20] Kearney AL, White KM (2016). Examining the psychosocial determinants of women's decisions to delay childbearing. Hum Reprod.

[21] Luk BH, Loke AY (2015). The impact of infertility on the psychological wellbeing, marital relationships, sexual relationships, and quality of life of couples: a systematic review. J Sex Marital Ther.

[22] Mumtaz Z, Shahid U, Levay A (2013). Understanding the impact of gendered roles on the experiences of infertility amongst men and women in Punjab. Reprod Health.

[23] Diamond R, Meyers M, Kezur D, Scharf CN, Weinshel M (1991). Couple therapy for infertility.

[24] Dimka RA, Dein SL (2013). The work of a woman is to give birth to children: cultural constructions of infertility in Nigeria. Afr J Reprod Health.

[25] Younesi SJ, Akbari-Zardkhaneh S, Behjati Ardakani Z (2006). Evaluating stigma among infertile men and women in Iran. J Reprod Infertil.

[26] Alhassan A, Ziblim AR, Muntaka S (2014). A survey on depression among infertile women in Ghana. BMC Women's Health.

[27] Fahami F, Quchani SH, Ehsanpour S, Boroujeni AZ (2010). Lived experience of infertile men with male infertility cause. Iran J Nurs Midwifery Res.

[28] Gonzalez LO (2000). Infertility as a transformational process: a framework for psychotherapeutic support of infertile women. Issues Ment Health Nurs.

[29] Musa R, Ramli R, Yazmie AWA, Khadijah MBS, Hayati MY, Midin M (2014). A preliminary study of the psychological differences in infertile couples and their relation to the coping styles. Compr Psychiatry.

[30] Cizmeli C, Lobel M, Franasiak J, Pastore LM (2013). Levels and associations among self-esteem, fertility distress, coping, and reaction to potentially being a genetic carrier in women with diminished ovarian reserve. Fertil Steril.

[31] Anokye R, Acheampong E, Mprah WK, Ope JO, Barivure TN (2017). Psychosocial effects of infertility among couples attending St.Michael's Hospital, Jachie-Pramso in the Ashanti Region of Ghana. BMC Res Notes.

[32] Kato A, Fujimaki Y, Fujimori S, Isogawa A, Onishi Y, Suzuki R (2016). Association between self-stigma and self-care behaviors in patients with type 2 diabetes: a cross-sectional study. BMJ Open Diabetes Res Care.

[33] Ceballo R, Graham ET, Hart J (2015). Silent and infertile: an intersectional analysis of the experiences of socioeconomically diverse African American women with infertility. Psychol Women Q.

[34] Ranjbar F, Behboodi-Moghadam Z, Borimnejad L, Ghaffari SR, Akhondi MM (2015). Experiences of infertile women seeking assisted pregnancy in Iran: a qualitative study. J Reprod Infertil.

[35] Ried K, Alfred A (2013). Quality of life, coping strategies and support needs of women seeking Traditional Chinese Medicine for infertility and viable pregnancy in Australia: a mixed methods approach. BMC Women's Health.

[36] Kabeer N (1999). Resources, agency, achievements: Reflections on the measurement of women's empowerment. Dev Change.

[37] Kiesswetter M, Marsoner H, Luehwink A, Fistarol M, Mahlknecht A, Duschek S (2020). Impairments in life satisfaction in infertility: Associations with perceived stress, affectivity, partnership quality, social support and the desire to have a child. Behav Med.

[38] Dennis CL (2003). Peer support within a health care context: a concept analysis. Int J Nurs Stud.

[39] Alavi NM, Alami L, Taefi S, Gharabagh GS (2011). Factor analysis of selftreatment in diabetes mellitus: a cross-sectional study. BMC Public Health.

